# The adult prevalence of HIV in Zambia: results from a population based mobile testing survey conducted in 2013–2014

**DOI:** 10.1186/s12981-015-0088-1

**Published:** 2016-01-19

**Authors:** Pascalina Chanda-Kapata, Nathan Kapata, Eveline Klinkenberg, Ngosa William, Liwewe Mazyanga, Katoba Musukwa, Elizabeth Chizema Kawesha, Felix Masiye, Peter Mwaba

**Affiliations:** Department of Disease Surveillance, Control and Research, Ministry of Health, Lusaka, Zambia; Center of Tropical Medicine and Travel Medicine, Department of Infectious Diseases, Academic Medical Centre, University of Amsterdam, Amsterdam, Netherlands; Department of Epidemiology and Disease Control, Ministry of Community Development, Mother and Child Health, Lusaka, Zambia; KNCV Tuberculosis Foundation, The Hague, Netherlands; Department of Global Health, Academic Medical Center, Amsterdam Institute for Global Health and Development, Amsterdam, The Netherlands; World Health Organisation, Lusaka, Zambia; Virology Laboratory, University Teaching Hospital, Lusaka, Zambia; Department of Economics, University of Zambia, Lusaka, Zambia

**Keywords:** Mobile testing, HIV/AIDS, Population survey, Infectious diseases, Zambia, Tuberculosis, Epidemiology

## Abstract

**Objective:**

To estimate the adult prevalence of HIV among the adult population in Zambia and determine whether demographic characteristics were associated with being HIV positive.

**Methods:**

A cross sectional population based survey to asses HIV status among participants aged 15 years and above in a national tuberculosis prevalence survey. Counselling was offered to participants who tested for HIV. The prevalence was estimated using a logistic regression model. Univariate and multivariate associations of social demographic characteristics with HIV were determined.

**Results:**

Of the 46,099 individuals who were eligible to participate in the survey, 44,761 (97.1 %) underwent pre-test counselling for HIV; out of which 30,605 (68.4 %) consented to be tested and 30, 584 (99.9 %) were tested. HIV prevalence was estimated to be 6.6 % (95 % CI 5.8–7.4); with females having a higher prevalence than males 7.7 % (95 % CI 6.8–8.7) versus 5.2 % (95 % CI 4.4–5.9). HIV prevalence was higher among urban (9.8 %; 95 % CI 8.8–10.7) than rural residents (5.0 %; 95 % CI 4.3–5.8). The risk of HIV was double among urban dwellers than among their rural counterparts. Being divorced or widowed was associated with a threefold higher risk of being HIV positive than being never married. The risk of being HIV positive was four times higher among those with tuberculosis than those without tuberculosis.

**Conclusions:**

HIV prevalence was lower than previously estimated in the country. The burden of HIV showed sociodemographic disparities signifying a need to target key populations or epidemic drivers. Mobile testing for HIV on a national scale in the context of TB prevalence surveys could be explored further in other settings.

## Background

About 38.1 million people have become infected with HIV and 25.3 million people have died of AIDS related illness [1, 2]. An estimated 36.9 (34.3–41.4) million people were living with HIV in 2014 with a global prevalence of 0.8 % [2, 3] Sub-Saharan Africa accounts for 70 percent of the global burden with an estimated 25.8 million people living with HIV [1, 2]. About half (51 %) of all people living with HIV do not know that they have the virus [2, 4].

Mobile HIV voluntary counselling and testing (MVCT) has begun to be implemented throughout Sub-Saharan Africa. The benefits of this approach include accessing first time testers, hard to reach populations such as men, rural populations and individuals at higher risk of HIV infection [5]. Same-day HIV testing in community settings seems to be acceptable in Sub-Saharan Africa [6]. Barriers to HIV testing are often logistic and can be overcome with community-based strategies [7]. These strategies require refining so as to address the needs of those not using mobile testing services [7]. A study in rural Kwazulu Natal in South Africa compared users of home and mobile VCT HIV testing and found that both modalities have an important role to play in reaching different populations and achieving high detection rates [8]. Easily accessible MVCT services combined with community mobilization programmes and psychosocial support after testing could increase rates of HIV testing and diagnosis, reduce individual risk behaviors, improve reproductive health decision making, increase access to treatment, reduce HIV/AIDS-related stigma and discrimination, and ultimately lower HIV incidence [9–11]. MVCT could also help to expand knowledge of personal HIV infection status [9–11]. MVCT has been promoted as a means of reaching populations with limited access to HIV testing, and has been effective in attracting large numbers of new testers in countries such as Tanzania, Zimbabwe and Cameroon [12].

Population surveys have been conducted in Zambia since 1992 to provide demographic and health indicators. Surveys done in 2001, 2007 and 2013/2014 were designed to measure HIV prevalence in Zambia [13–15]. The trends for HIV prevalence measured by the Demographic Health Surveys (DHS) suggest a reducing burden/trend from 16 % in 2001 to 14 % in 2007 and 13 % in 2014 among adults aged 15–49 years old. However, the method of screening participants has been through household testing.

The feasibility of large scale mobile testing for HIV has not been tested at a population level in Zambia. We present here latest results of the prevalence of HIV using a mobile testing approach in the context of the national TB prevalence survey. The socio-economic determinants of HIV are also discussed. This is the largest study to measure the HIV prevalence at a national level in Zambia.

## Methods

### Study design

This was a cross sectional evaluation of the prevalence of HIV among adults aged 15 years and above who were participants in a national Tuberculosis Prevalence Survey that was conducted from 2013 to 2014. The sample was drawn from 66 primary sampling units (clusters). The clusters were census supervisory areas (CSAs) from both rural and urban areas. The average size of each CSA was 700–1200 participants. Clusters were selected using a two-stage sampling strategy.

### HIV testing set up and algorithm

All survey participants were offered HIV testing after they were counselled and consented. Survey nurses who were certified in counselling skills conducted pre- and post- test HIV counselling. The signed certificate of consent for HIV testing form was separate from the general TB survey consent form. The consent form also indicated whether the participant wished to know the results of the HIV test or not.

Rapid HIV testing was performed on site on an opt-out basis. The HIV testing station was located at the exit of the TB survey related procedures at the main survey site.

HIV test results were communicated in a private area at the central site to ensure privacy and confidentiality. When the test was negative, the participant was given the result as not-infected. When the test was positive, the participant was referred to the nearest voluntary counselling and testing (VCT) facility for further evaluation and management. Further confirmation for HIV positive participant was also done on site. Individuals who tested positive for HIV were referred for further management to a nearby health facility. Pre-test and post-test counselling was provided to all participants who choose to know their results.

### HIV testing algorithm

For the purpose of the survey, HIV testing and counselling was conducted in accordance with national algorithm. Pre-test counselling was done prior to testing. Participants who chose not to know the results still provided a blood sample for HIV testing for the purpose of the survey only.

Following the screening interview by the nurse counsellor, participants that consented to HIV testing had blood collected through a finger prick and placed directly onto the screening test strip (Determine^®^). Participants, who did not wish to know their HIV test result, exited the survey after this stage and were thanked for participating.

The process was different for participants who wanted to know their HIV results. Post-test counselling was done after the initial test results were available. If the test was non-reactive, a negative result was communicated to the participant. However, if the test was reactive, the participant was asked to give a second blood sample from another finger prick for a confirmatory test (Uni-Gold™ Recombigen^®^). If the second test was reactive, the participant was informed of his/her positive status, appropriately counselled and referred for care at the nearest health facility for case management and follow up. If the second test was non-reactive, the result was considered indeterminate and the participant was counselled and advised to visit the nearest health facility for a further follow-up testing after 6 weeks, in accordance with national guidelines.

Consent status and HIV test results were documented on personal digital assistants (PDAs) which contained pre-programmed survey questionnaires. The HIV related questionnaire was linked to the TB survey related questionnaires/outcomes and socio-economic questionnaire using a bar code which was programmed as a unique personal identifier code (UPIC). No names or identifiers were collected in the data bases to ensure confidentiality of the participants.

### Ethical considerations

The study protocol was cleared by the University of Zambia Biomedical Research Ethics Committee (UNZABREC) No: 020-08-12. Authorisation to conduct the survey was sought in line with the existing national policies and guidelines at national, provincial and district levels. The Institutional Review Board (IRB) approved this consent procedure. All the consent or assent forms were recorded on standard forms which were developed for the study and these were filed in lockable cabinets at the end of each cluster operation and stored in central archives as per national requirements.

### Data analysis and modelling approach

Data was analysed in STATA version 13. For background characteristics, frequencies and proportions were generated. In order to measure the association between HIV status and participants’ background variables; binary logistic regression was done for bivariate variables (sex and setting); multinomial logistic regression for polychotomous variables (education level, marital status and wealth quintile).

Estimation of the HIV prevalence was initially done using a simple cluster level model whereby the prevalence rates were calculated at cluster level and then combined to one single point estimate with confidence boundaries. The second step was an individual level model analysis whereby logistic regression models were applied restricted to participants with complete outcomes only, so called complete case analysis. Individuals with missing data on outcome, i.e., HIV test result were excluded.

The third step was a model whereby all eligible individuals irrespective of participation were included and through multiple missing value imputation outcomes were imputed for all eligible participants. This method uses a logistic regression model with robust standard errors (RSE), with missing value imputation for survey non-participants as well as participants, and includes all individuals who were eligible for the survey in the analysis. This method allows for both the clustering in the sampling design and the uncertainty introduced by imputation of missing values when estimating the 95 % CI for the prevalence of HIV.

## Results

**Fig. 1:**
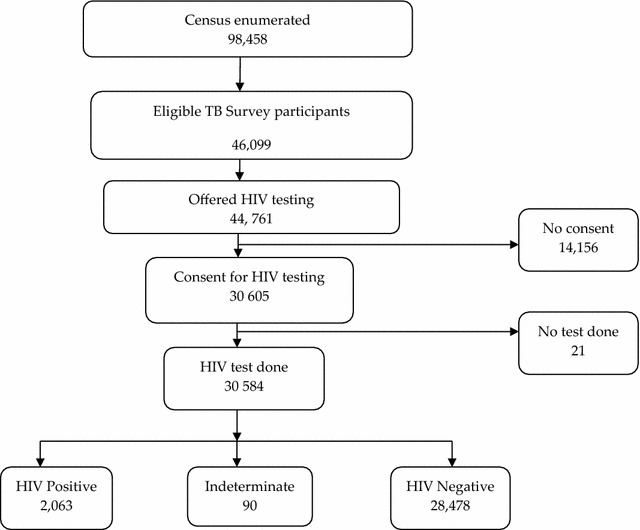
Flow diagram of survey participants offered HIV counselling and testing

Of the 46,099 individuals who were eligible, 44,761 (97.1 %) underwent HIV counselling and testing; out of which 30,605 (68.4 %) consented to be tested for HIV and 30,584 (99.9 %) were tested. Of those who consented, 21 individuals had no HIV test performed and hence were excluded from further analysis. A total of 30,331 (99.1 %) individuals who were tested indicated that they wanted to know their test results. The flow of participants is summarised in Fig. 1.

Of the 30,584 participants who consented and were tested, two-thirds were from a rural setting; more than half were female and a third were aged between 15 and 24 years. The majority (46.2 %) had at least primary education; more than half were married or living as married and about 22 % were from the highest wealth quintile as shown in Table 1.

**Table 1:** Background characteristics of those who were tested for HIV

Variable	Frequency	Percentage (%)	95 % confidence interval
Overall	30,584		
Setting			
Rural	21,052	68.8	68.3–69.4
Urban	9533	31.2	30.7–31.7
Sex			
Male	12,943	42.3	41.8–42.9
Female	17,641	57.7	57.1–58.2
Age group			
15–24	9719	31.8	31.3–32.3
25–34	7219	23.6	23.1–24.1
35–44	5466	17.9	17.4–18.3
45–54	3591	11.7	11.4–12.1
55–64	2455	8.0	7.7–8.3
65+	2134	7.0	6.7–7.3
Education level			
None	2655	8.7	8.4–9
Primary	14,128	46.2	45.6–46.8
Secondary	12,675	41.4	40.9–42
Tertiary	1114	3.6	3.4–3.9
Unknown	12	0.0	0
Marital status			
Never	8372	27.4	26.9–27.9
Married	17,800	58.2	57.6–58.8
Divorced	2080	6.8	6.5–7.1
Widowed	2332	7.6	7.3–7.9
Wealth quintile			
Lowest	4830	18.1	17.6–18.6
Second lowest	4844	18.2	17.7–18.6
Middle	5539	20.8	20.3–21.3
Fourth	5610	21.0	20.5–21.5
Highest	5847	21.9	21.4–22.4

Overall, 2063 (6.8 %) of those tested were found to be HIV positive; while 90 (0.3 %) had an indeterminate result and 28,431 (93.0 %) were negative. Cluster level analysis of the HIV prevalence resulted in an overall prevalence of 7.2 % (95 % CI 6.1–8.3). Missing value imputation modelling approach showed similar results, 6.6 % (95 % CI 5.8–7.4). The prevalence of HIV by participant background characteristics is presented in Table 2. The prevalence of HIV among the urban residents was almost double that of the rural residents (5.0 versus 9.8 %). By gender, the prevalence was higher among the female than the male participants (7.7 versus 5.2 %). The prevalence of HIV was similar by education level. The HIV prevalence was twice and more than thrice in the married and widowed or divorced than the never married respectively.

**Table 2:** Estimated HIV prevalence by setting, sex, education level and marital status of participants

Variable	Prevalence estimate (%)	95 % CI
Overall modelled HIV prevalence	6.6	5.8–7.4
By setting		
Rural	5.0	4.3–5.8
Urban	9.8	8.8–10.7
By sex		
Male	5.2	4.4–5.9
Female	7.7	6.8–8.7
By education level		
No schooling	5.9	4.8–7.0
Primary school	7.1	6.0–8.3
Secondary school	7.0	6.0–7.9
Tertiary education	7.3	5.7–9.0
By marital status		
Never married	3.6	3.0–4.3
Currently/living as married	6.9	5.9–8.0
Divorced/separated	15.1	12.9–17.3
Widowed	11.7	10.0–13.4

**Fig. 2:**
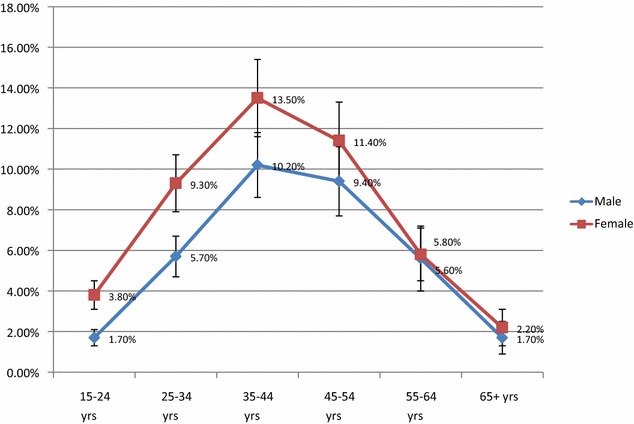
Estimated HIV prevalence by age and sex with uncertainty bounds

Analysis of the HIV prevalence by sex and age showed that between 15 and 44 years, there were gender differences in prevalence; with females having a higher prevalence than males (Fig. 2). From 45 years onwards, the HIV prevalence was similar by gender. Additionally, the HIV Prevalence increased by age for both male and female peaking in the age band 35–44 years; thereafter the HIV prevalence steadily reduced with increasing age. The HIV prevalence was lowest among males aged 15–24 years (1.7 %) peaking at 10.2 % among those aged 35–44 years but declining thereafter to 1.7 % among those aged 65 years and above. For females, the HIV prevalence among those aged 15–24 years was 3.8 %; peaking at 13.5 % among those aged 35–44 years and the lowest prevalence was 2.2 % among those aged 65 years and above (see Fig. 2).

**Fig. 3:**
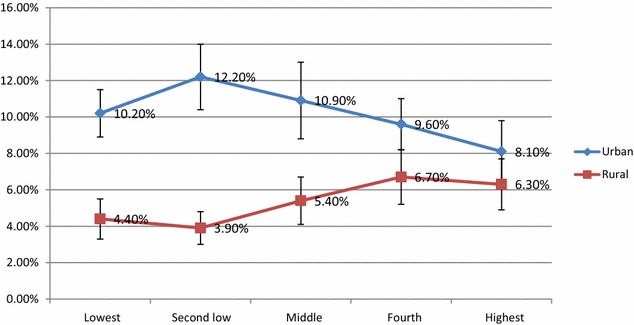
Estimated HIV prevalence by wealth quintile for urban and rural areas with uncertainty bounds

HIV prevalence was generally higher in the higher wealth quintiles compared to lower wealth quintile (4.3 versus 8.9 %). However, when disaggregating the wealth quintiles by setting, the HIV Prevalence in the urban was higher in the lower wealth quintiles than in the higher wealth quintiles (10.2 versus 8.1 %). Whilst in the rural setting, the HIV prevalence was higher in the higher wealth quintiles than in the lower wealth quintiles (6.3 versus 4.4 %) as shown in Fig. 3.

Table 3 shows that the prevalence of HIV was four times higher among bacteriological confirmed or smear positive TB cases than those without TB. The prevalence of HIV was similar by non-tuberculous mycobacteria status.

**Table 3:** Estimated HIV prevalence by TB status

	Estimate expressed as a percentage (%)	95 % CI
Overall	6.6	5.8–7.4
By bacteriological TB status		
No bacteriological confirmed TB	6.5	5.7–7.3
Bacteriological confirmed TB	26.9	18.0–35.8
By smear TB status		
Smear negativeTB	6.8	5.9–7.7
Smear positive TB	28.4	16.7–40.1
By non-tuberculous mycobacteria status		
No symptomatic NTM	6.9	6.0–7.8
Symptomatic NTM	8.9	6.4–11.4

The results of the univariate and multivariate logistic regressions are shown in Table 4. In the univariate analysis, the likelihood of being HIV positive was 1.8 times higher among participants from the urban compared to the rural areas (p = 0.000). Males were 0.9 times (p = 0.000) less likely than the females of being HIV positive. The differences in the likelihood of being HIV positive by age group, education level, marital status and wealth quintiles were significant (p = 0.000). In the multivariate analysis, the urban participants were 2.0 times more likely than the rural to be HIV positive; the males were 0.7 times less likely of being HIV positive than females. By age group, those aged 35–44 years were 4.7 times more likely to be HIV positive than those aged 15–24 years. The married were 0.7 less likely to be HIV positive than the never married (p = 0.000). The divorced and widowed were 1.5 and 1.4 times more likely to be HIV positive than the never married respectively (p = 0.000). The participants in the highest wealth quintile were 2.5 times more likely to be HIV positive than those from the lowest wealth quintile (p = 0.000).

**Table 4:** Univariate and multivariate association for HIV positivity and participant background characteristics

Variable	Univariate			Multivariate		
	OR	95 % CI	P value	OR	95 % CI	P value
Setting						
Rural	ref			ref		
Urban	1.8	1.7–1.9	0.000	2.0	1.9–2.2	0.000
Sex						
Male	0.9	0.9–1.0	0.000	0.7	0.6–0.7	0.000
Female	ref			ref		
Age group						
15–24	ref			ref		
25–34	1.0	1.0–1.1	0.679	3.0	2.6–3.5	0.000
35–44	1.3	1.2–1.3	0.000	4.7	4.1–5.5	0.000
45–54	1.2	1.1–1.3	0.000	4.1	3.5–4.8	0.000
55–64	1.2	1.0–1.2	0.004	2.1	1.7–2.6	0.000
65+	1.4	1.3–1.5	0.000	0.7	0.5–1.0	0.040
Education level						
None	ref					
Primary	0.81	0.8–0.9	0.000	1.2	1.0–1.4	0.035
Secondary	0.9	0.8–1.0	0.002	1.2	1.0–1.4	0.120
Tertiary	1.0	0.9–1.1	0.773	1.1	0.8–1.5	0.489
Marital status						
Never	ref					
Married	0.9	0.9–1.0	0.000	0.7	0.6–0.8	0.000
Divorced	1.2	1.1–1.3	0.000	1.5	1.3–1.7	0.000
Widowed	1.5	1.4–1.6	0.000	1.4	1.2–1.5	0.000
Wealth quintile						
Lowest	ref					
Second lowest	1.0	0.9–1.0	0.273	1.2	1.0–1.5	0.029
Middle	1.2	1.1–1.3	0.000	2.0	1.7–2.4	0.000
Fourth	1.5	1.4–1.6	0.000	2.4	2.0–2.9	0.000
Highest	1.7	1.6–1.8	0.000	2.5	1.9–2.7	0.000

The sub-national analyses showed variations in the HIV prevalence with the highest being Western Province at 11.9 % (95 % CI 8.0–15.8) while Muchinga Province had the lowest at 1.5 % (95 % CI1.5–1.6) as shown in Table 5.

**Table 5:** Prevalence of HIV by province

	Prevalence estimate (%)	95 % CI
Overall	6.8	5.6–7.9
By province		
Central	5.2	3.6–6.8
Copperbelt	8.3	6.6–9.9
Eastern	3.4	1.7–5.1
Luapula	5.2	2.4–8.0
Lusaka	11.3	9.4–13.2
Muchinga	1.5	1.5–1.6
Northern	3.0	1.3–4.6
North western	5.9	3.8–8.0
Southern	8.1	4.9–11.2
Western	11.9	8.0–15.8

## Discussion

In a population based survey of participants in a national TB prevalence survey, the estimated prevalence of HIV was found to be 6.6 % (95 % CI 5.8–7.4). There were socio-demographic variations in the burden and risk of HIV. This study represents the largest data set to estimate the population based prevalence of HIV in Zambia. This is also the first study to test the national sero-prevalence of HIV using mobile population based testing on a national level as opposed to the DHS where testing is done at household level. Additionally, this is the first national TB prevalence survey conducted in which HIV testing was offered to all eligible survey participants. The participant response rate reported in the study is similar to what has been reported in other national surveys in Africa [16].

The burden of HIV was higher in the urban than rural individuals; however, the participants in the lower wealth quintiles (“urban poor”) had a higher HIV burden than those from highest wealth quintiles in the urban areas. In rural areas however, this pattern was reversed with the burden of HIV being higher among participants from the higher wealth quintiles than those from the lower wealth quintiles. The disparities in HIV burden by socioeconomic status disaggregated by rural/urban setting seem to suggest that HIV is not merely a disease of the poor but rather that it depends on the context of the sub-population being looked at. There is need to investigate and clearly understand the locally relevant risk factors so that effective strategies can be implemented for the urban, females, those aged 35–44, widowed or divorced and the less educated. Risk reduction strategies targeting these high risk groups need to be implemented along with other HIV programmes. The risk and burden of HIV is higher in an age group which is supposed to be economically active and hence emphasize the larger economic consequence of the disease.

The groups with a high risk HIV prevalence found in this study are similar to what has been found in national household level HIV testing studies in Africa, Zambia inclusive [16]. This therefore signifies that mobile testing approaches on a national level may provide alternative reliable estimates of the burden of HIV.

This study has shown that provider initiated mobile testing and counselling has the potential to complement access to HIV testing as has been supported by other studies [17, 18]. Additionally, participants who didn’t know their HIV status prior to the survey were linked to the nearest health facility for case management in line with national guidelines. The approach to test people outside of the health facility created an opportunity for testing a large number of people (more than 30, 000) within 1 year and thus it is possible that mobile counselling and testing for HIV in communities can provide a faster approach to achieve universal counselling and testing for HIV. Such community based testing approaches have the potential to increase HIV testing and counselling and should be adopted as routine public health programs where feasible [19]. Programs to address the high burden among the urban poor, widows and those co-infected with TB are some of the critical areas of focus for HIV burden and risk reduction post-2015. Such programs should target to provide ongoing provider initiated counselling and testing in the communities among these groups with strong linkages to health facility for follow up. Some strategies could include setting up mobile testing units in urban areas, implementing HIV related programs in schools, churches and other local community groups [20–22]. Increasing the number of people tested has the potential to also increase the number of people put on anti-retroviral treatment (ART) [23].

The prevalence of HIV found in this survey may have been affected by selection bias due to the non-participation of some eligible individuals. The characteristics of those who declined to test have been documented elsewhere [24]; rates of decliners were higher among those aged 15–24, from the highest wealth quintile, female and married. However, a review of national population based studies from eight African countries showed that non-response is unlikely to significantly affect the estimates of the burden of HIV in national HIV sero-prevalence surveys [16].

Finally, this study has shown that it is feasible to perform mobile HIV counselling and testing in the context of national Tuberculosis prevalence surveys. This linkage has the potential to reduce the resource burden required to conduct these surveys separately. However, careful planning is required to ensure high participation rates so as to reduce selection bias.

## Conclusions

HIV prevalence in Zambia is characterized by sociodemographic disparities. Mobile testing for HIV has the potential to increase the number of people tested. Programs to address the high burden among the urban poor, widows and those co-infected with TB are some of the critical areas of focus for HIV burden and risk reduction post-2015. Conducting HIV sero-prevalence testing within the national TB prevalence surveys could be explored further in other settings.
